# Relationship of Aortopulmonary Collaterals and Pulmonary Artery Development During Staged Single Ventricle Reconstruction

**DOI:** 10.1007/s00246-024-03484-y

**Published:** 2024-04-30

**Authors:** Helena Staehler, Thibault Schaeffer, Stanimir Georgiev, Melvin Schmiel, Christoph Stern, Chiara Di Padua, Nicole Piber, Alfred Hager, Peter Ewert, Jürgen Hörer, Masamichi Ono

**Affiliations:** 1 Department of Congenital and Pediatric Heart Surgery, German Heart Center Munich, Technische Universität München, Munich, Germany; 2 Division of Congenital and Pediatric Heart Surgery, University Hospital of Munich, Ludwig-Maximilians-Universität in Munich, Munich, Germany; 3 Europäisches Kinderherzzentrum München, Munich, Germany; 4 Department of Congenital Heart Disease and Pediatric Cardiology, German Heart Center Munich at the Technical University of Munich, Munich, Germany; 5 Department of Cardiovascular Surgery, German Heart Center Munich, Technische Universität München, Munich, Germany

**Keywords:** Single ventricle, Pulmonary artery, Total cavopulmonary connection, Bidirectional cavopulmonary shunt, Aortopulmonary collaterals

## Abstract

To evaluate the relationship of aortopulmonary collaterals and the development of central pulmonary arteries during staged palliation. A total of 287 patients, who underwent staged palliation with bidirectional cavopulmonary shunt and total cavopulmonary connection between 2008 and 2019, had available angiography. Pulmonary artery index was calculated using pulmonary angiography as described by Nakata and colleagues. Aortopulmonary collaterals were observed in 47 (16%) patients at stage II palliation, in 131 (46%) at total cavopulmonary connection, and afterwards in 49 (7%). The interventional closure of aortopulmonary collaterals was performed before stage II in 12 (4%) patients, before Fontan completion in 38 (13%), and afterwards in 39 (14%). Presence of aortopulmonary collaterals before stage II was not associated with the pulmonary artery index (129 vs. 150 mm^2^/m^2^, *p* = 0.176) at stage II. In contrast, aortopulmonary collaterals before the Fontan completion were associated with lower pulmonary artery index (154 vs. 172 mm^2^/m^2^, *p* = 0.005), and right pulmonary artery index (99 vs. 106 mm^2^/m^2^, *p* = 0.006). Patients who underwent interventional closure of aortopulmonary collaterals before total cavopulmonary connection had lower pulmonary artery index (141 vs. 169 mm^2^/m^2^, *p* < 0.001), lower right pulmonary artery index (93 vs. 106 mm^2^/m^2^, *p* = 0.007), and left pulmonary artery index (54 vs. 60 mm^2^/m^2^, *p* = 0.013) at Fontan completion. The presence of aortopulmonary collaterals did not influence pulmonary artery size by the time of stage II. However, presence of aortopulmonary collaterals was associated with under-developed pulmonary arteries at Fontan completion, especially in patients who needed interventional closure of aortopulmonary collaterals.

## Introduction

The clinical significance of aortopulmonary collaterals (APCs) in patients with univentricular heart remains controversial. APCs represent an unpredictable source of pulmonary blood flow in patients with univentricular heart and are observed at each stage of surgical reconstruction toward Fontan circulation, as well as after the Fontan completion [1]. While APCs increase oxygen saturation in the short-term, they create a hemodynamic burden in single ventricle physiology by shunting up to 30–50% of total systemic blood flow to the lungs [2]. Whereas earlier studies are still discordant on the influence of APCs, more recent studies show that increased APCs are associated with adverse early post-Fontan outcomes [2, 3]. Many patients acquire a large amount of APC flow after BCPS which ultimately influences the occurrence of adverse events after TCPC [4]. There have been a number of reports of adverse events which have been attributed to the presence of APCs and the amount of APC flow. These include increased chest drain volume, prolonged hospitalization and postoperative recovery, and impaired neurodevelopmental outcome [5–7].

While the overall effects of APCs have been widely described, the precise mechanisms leading to them are not well understood. One study suggests that the APC flow is related to small pulmonary artery size, implying that small pulmonary arteries may stimulate the development of APCs [8]. Furthermore, we have only recently been able to show that lower Nakata-Index is associated with the development of APCs [1].

However, the question remains whether APC development is influenced by pulmonary artery size or vice versa. In previous studies, we have investigated both the influence of APCs and the influence of pulmonary artery indices on the outcome after TCPC [1, 9]. The aim of this study was to investigate the relationship between these two factors and evaluate the impact of APCs on the pulmonary artery development during staged palliation.

## Patients and Methods

### Ethical Statement

The Institutional Review Board of the Technical University of Munich approved the study (approval number: 2023-638-S-SB on 7th December 2023) and waived the need for informed consent from the patients who were retrospectively analyzed in the study.

### Patients

We reviewed a total of 287 patients who met the following inclusion criteria: having undergone BCPS and TCPC at the German Heart Center Munich between 2008 and 2019, and availability of angiography data. During this study period, non-fenestrated extracardiac TCPC was our standard and fenestration was made only in selected patients. Patients who underwent fenestration at TCPC, those without available angiography data, and those without available data of current clinical status were excluded. The PA index before BCPS and TCPC were calculated using angiography as described by Nakata and colleagues [10]. The right and left PA index was calculated by dividing the cross-sectional area of each PA branch by the body surface area. Medical records included baseline morphology, in-hospital and out-hospital records, and cardiac catheterization data using electronic and paper chart reviews. The patients regularly obtained outpatient follow-up with pediatric cardiologists. Follow-up data from the time of Fontan completion until the last record of the patient were regularly updated using our institutional single ventricle patient database.

### Operative Techniques

The surgical techniques for BCPS and TCPC are described in our previous reports [11, 12]. BCPS was performed through median sternotomy with cardiopulmonary bypass and standard bicaval cannulation. Cardioplegic arrest was only applied for patients who required intracardiac procedures. The azygous vein was ligated and transected in all patients. Ante-grade pulmonary blood flow was maintained at BCPS only in selected patients. The PA configuration was evaluated by cardiac catheterization before BCPS in all patients and concomitant PA patch enlargement was performed, if needed. TCPC was always performed on cardiopulmonary bypass using an extracardiac polytetrafluoroethylene graft. In this study period, fenestration was used only for high-risk patients [9]. Surgical closure of APCs at the time of operation included right internal mammary artery (RIMA) and/or left internal mammary artery (LIMA) closure. After the median sternotomy, RIMA and/or LIMA was prepared, closed and divided using clips at the most proximal and most distal site [1].

### APC Detection and Hemodynamic Interpretation by Angiography

The presence of APCs was determined by the finding of angiogram as described by earlier reports [1, 13]. We reviewed pulmonary angiograms for areas of washout in all patients. Selective angiography of the brachiocephalic, subclavian, and internal thoracic arteries, as well as aortography was then performed. APCs were defined as arising from the arterial circulation if they had a discrete angiographically identifiable origin, supplied the lung parenchyma, and obstructed the pulmonary arteries or veins or both. The diameter of each identifiable collateral vessel was estimated by comparing the vessel diameter to the known diameter of the catheters depicted in the angiogram. Angiograms in which collateral vessels were visualized but the origin or distribution of blood flow was unclear were not included in the analysis. As we assume that APCs might elevate the pulmonary artery pressure and interfere with the development of central pulmonary arteries, we tended to perform interventional embolization of APCs in most cases when technically feasible. When we decided to intervene, different factors were taken into account-pulmonary artery pressure, arterial saturation, opacification of the distal pulmonary arterial bed or pulmonary veins during selective injection into the collateral and the size of the APCs were judged subjectively. Techniques and devices used for closure were chosen based on the size and morphology of the collateral, with coils being the most common device used. The effect of the embolization was evaluated using angiography at the end of the procedure.

### Statistical Analysis

Categorical variables are presented as absolute numbers and percentages. Continuous variables are expressed as medians with interquartile ranges (IQR). The comparison of PA index between the patients with and without APCs was performed using non-paired *t* test. The comparison between PA index values before BCPS and before TCPC was performed by paired *t* test. Data normality was assessed by Kolmogorov–Smirnov–Lilliefores-Test. Statistical significance was defined as *p* < 0.05. Data analysis and graphing were performed with the Statistical Package for the Social Sciences version 25.0 for Windows (IBM, Ehningen, Germany).

## Results

### Patients

Patient characteristics are displayed in Table 1. The most frequent diagnosis was hypoplastic left heart syndrome (HLHS) in 118 (41%) patients. Median age at BCPS and TCPC was 4.0 (IQR: 3.0–6.0) months and 2.0 (1.7–2.5) years, respectively. Median weight at BCPS and TCPC was 5.6 (3.2–13.9) kg and 12.2 (8.7–63.0) kg, respectively.

**Table 1:** Baseline characteristics of patients

Variables		*N* (%) or median (IQR)
Number of patients		287
Male Sex		182 (63.4)
Age at BCPS (months)		4.0 (3.0–6.0)
Weight at BCPS (kg)		5.2 (4.6–6.1)
Age at TCPC (years)		2.0 (1.7–2.5)
Weight at TCPC (kg)		11.4 (10.5–12.7)
ICU stay (days)		6 (4–8)
Hospital stay (days)		19 (13–27)
Primary Diagnosis		
	Hypoplastic left heart syndrome (HLHS)	118 (41.1)
	Univentricular heart (UVH)	52 (19.1)
	Tricuspid atresia (TA)	35 (12.2)
	Double inlet left ventricle (DILV)	26 (9.1)
	Pulmonary atresia and intact ventricular septum (PIVS)	16 (5.6)
	Congenitally corrected transposition of the great arteries (ccTGA)	14 (4.9)
	Unbalanced atrioventricular septal defect (UAVSD)	11 (3.8)
	Other Morphologies	15 (5.2)
Associated Anomalies		
	Transposition of the great arteries (TGA)	61 (21.3)
	Double outlet right ventricle (DORV)	36 (12.5)
	Coarctation of the aorta (CoA)	28 (9.8)
	Anomalous pulmonary venous drainage	24 (8.4)
	Anomalous systemic venous drainage	32 (11.1)
	Common atrioventricular valve (CAVV)	27 (9.4)
	Heterotaxy	21 (7.3)
Stage I Procedure		
	Norwood Procedure	158 (55.1)
	Sano shunt	71 (24.7)
	Modified Blalock Taussig Shunt	87 (30,.3)
	Aortopulmonary shunt	76 (26.4)
	PAB	22 (7.7)
Adverse events		
	ECMO	8 (2.8)
	Early death (< 30 days)	2 (0.7)
	Late death (> 30 days)	2 (0.7)

APCs were observed in 47 (16%) patients at BCPS, in 131 (46%) at TCPC, after TCPC in 49 (17%). Interventional closure of APCs was performed before BCPS in 12 (4%) patients, before TCPC in 38 (13%), and after TCPC in 39 (14%). Incidences of APCs are displayed in Table 2.

**Table 2:** Incidence of APCs

Variables		*N* (%)
Number of patients		287
Presence of APCs		160 (55.7)
Laterality		
	Right	154 (53.7)
	Left	111 (38.7)
Timing		
	Pre-BCPS	47 (16.4)
	After BCPS	13 (4.5)
	Pre-TCPC	131 (45.6)
	After TCPC	49 (17.1)

PA index did not change significantly from time of measurement before BCPS to before TCPC (149 (108–216) mm^2^/m^2^ to 162 (128–212) mm^2^/m^2^, *p* = 0.745). However, right PA index increased from 75 (57–116) mm^2^/m^2^ before BCPS to 103 (76–132) mm^2^/m^2^ before TCPC (*p* < 0.01). Left PA index decreased from 64 (44–100) mm^2^/m^2^ before BCPS to 59 (42–85) mm^2^/m^2^ before TCPC (*p* < 0.01).

### Impact of APCs on Pulmonary Artery Index

**Fig. 1:**
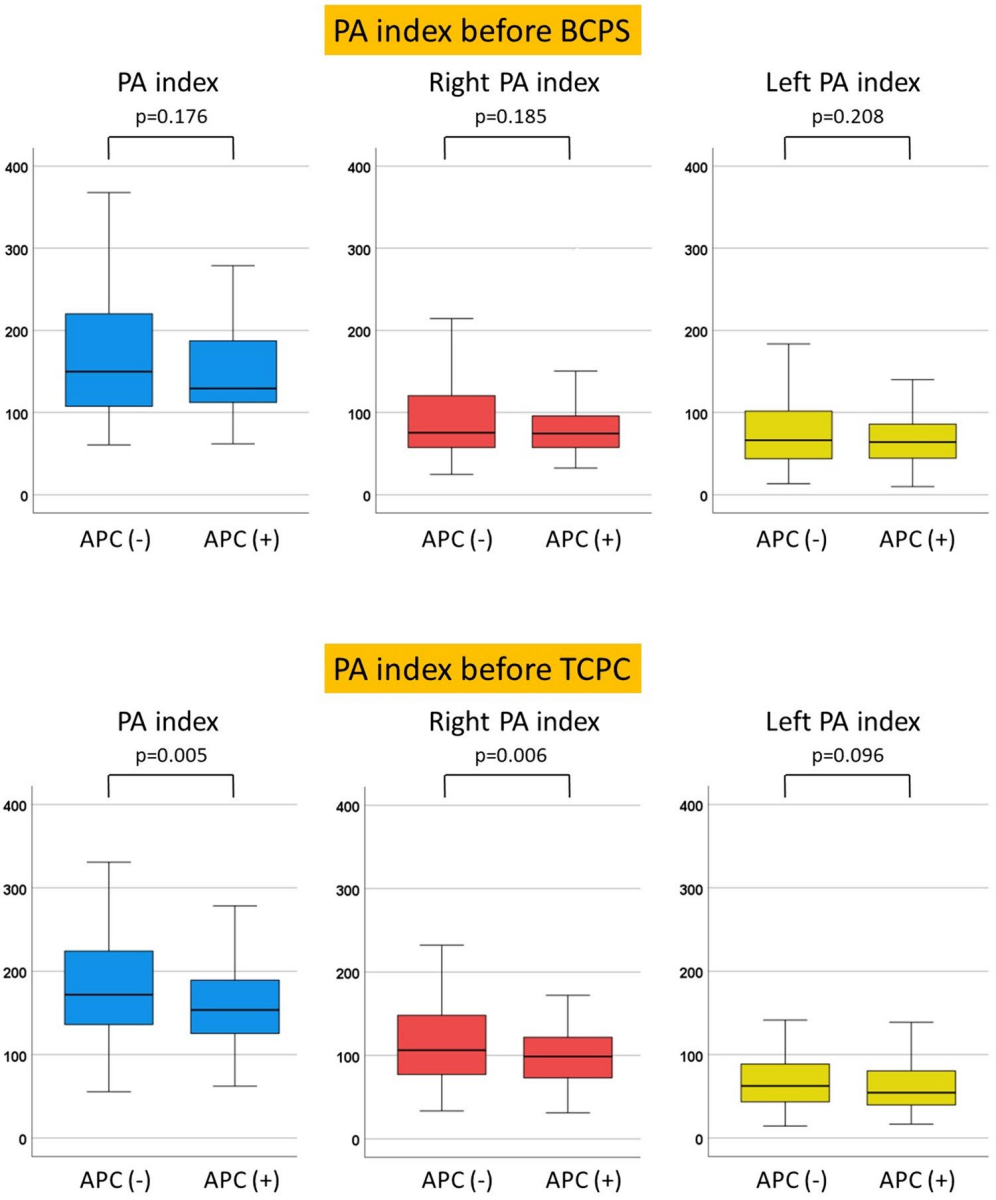
Comparison of PA index between patients with and without APCs before BCPS and TCPC

The presence of APCs prior to BCPS did not influence the PA index (129 vs. 150 mm^2^/m^2^, *p* = 0.176), or the right PA index (75 vs. 76 mm^2^/m^2^, *p* = 0.185), or the left PA index (64 vs. 66 mm^2^/m^2^, *p* = 0.208) at BCPS (Fig. 1).

On the contrary, observed APCs before TCPC were associated with lower PA index (154 vs. 172 mm^2^/m^2^, *p* = 0.005) and lower right PA index (99 vs. 106 mm^2^/m^2^, p = 0.006), compared to those patients who did not demonstrate APCs. Left PA index was similar in both groups (54 vs. 63 mm^2^/m^2^, *p* = 0.096) (Fig. 1).

**Fig. 2:**
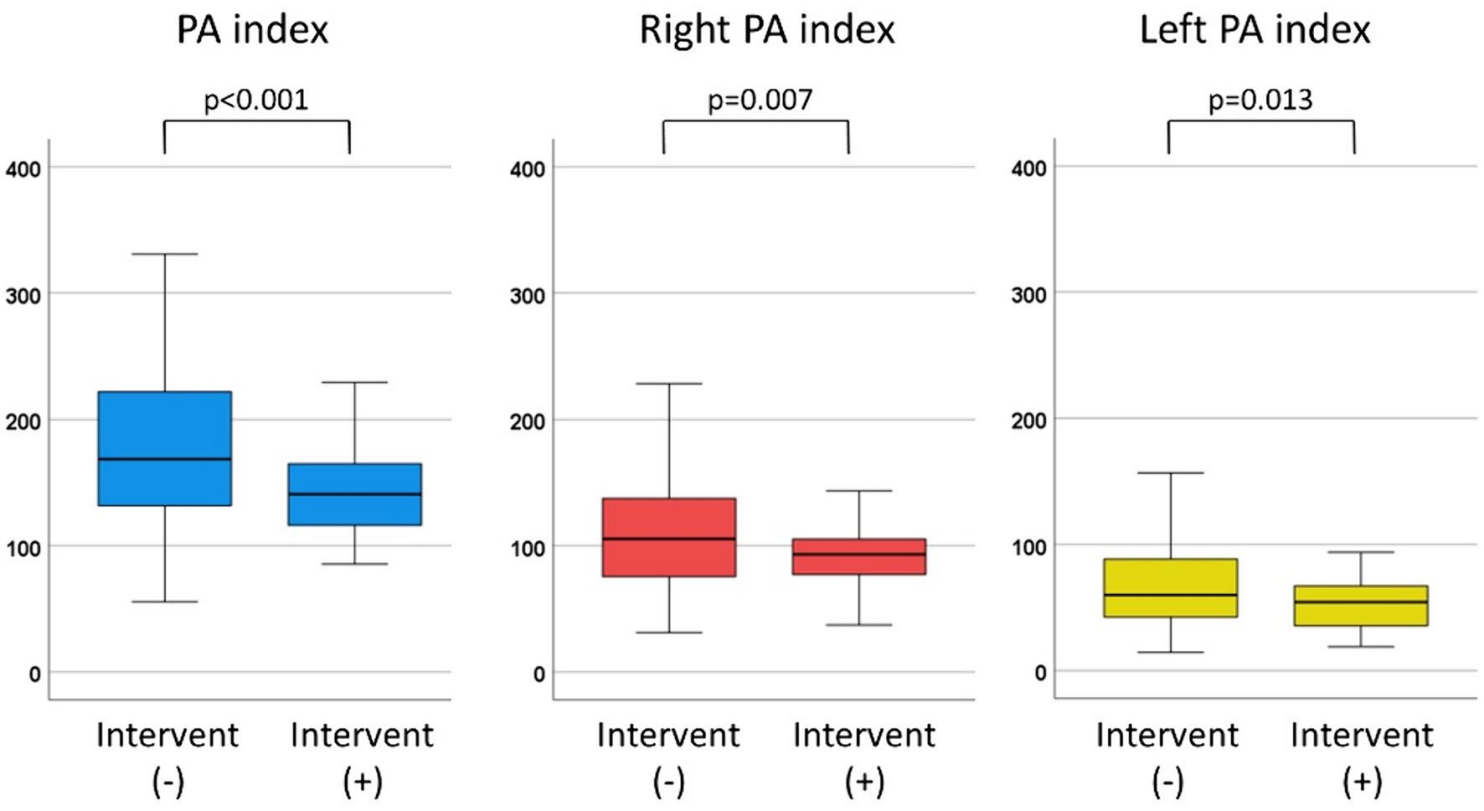
Comparison of PA index between the patients who underwent interventional APCs closure at the time of TCPC and those who did not

Patients who underwent interventional closure of APCs before TCPC had lower PA index (141 vs. 169 mm^2^/m^2^, *p* < 0.001), lower right PA index (93 vs. 106 mm^2^/m^2^, *p* = 0.007), and lower left PA index (54 vs. 60 mm^2^/m^2^, *p* = 0.013) at TCPC, compared to those who did not (Fig. 2).

### Change of PA Index in Patients with and Without APCs Between BCPS and TCPC

Patients were divided into two groups, those who demonstrated APC at the time of TCPC and those who did not. We then analyzed and compared changes in PA index between BCPS and TCPC separately for the two groups. Patients with APCs at TCPC had lower PA index at BCPS than those without (155 mm^2^/m^2^ vs. 191 mm^2^/m^2^) (*p* < 0.01). Also, left and right PA index, were lower in patients with APC at the time of BCPS (104 mm^2^/m^2^ vs. 85 mm^2^/m^2^ and 86 mm^2^/m^2^ vs. 72 mm^2^/m^2^). Even though overall PA index increased between the two procedures in the APC group (155 mm^2^/m^2^ to 164 mm^2^/m^2^), this observation could not be supported statistically (*p* = 0.114). However, right PA index increased significantly between BCPS and TCPS in patients with APCs (85 mm^2^/m^2^ to 101 mm^2^/m^2^, *p* < 0.001), whereas left PA index decreased during the observed time period (72 mm^2^/m^2^ to 65 mm^2^/m^2^, *p* = 0.047).

In comparison, patients without APCs showed greater PA index at BCPS (191 mm^2^/m^2^). No significant increase in right PA index could be observed (104 mm^2^/m^2^ to 113 mm^2^/m^2^) (*p* = 0.089). However, left PA index decreased significantly between BCPS and TCPC in patients without APCs (86 mm^2^/m^2^ to 71 mm^2^/m^2^) (*p* < 0.001). The described results are displayed in Table 3.

**Table 3:** Change of PA index in patients with and without APCs at TCPC

Variables	At BCPS	At TCPC	*p*-value
Comparison of APC at TCPC			
Patients without APC			
PAI	191 ± 101	185 ± 72	0.434
RPAI	104 ± 57	113 ± 50	0.089
LPAI	86 ± 52	71 ± 33	< 0.001
Patients with APC			
PAI	155 ± 73**	164 ± 57**	0.114
RPAI	85 ± 45**	101 ± 37*	< 0.001
LPAI	72 ± 42*	65 ± 37	0.047
Comparison of APC interventions at TCPC			
Patients without APC Intervention			
PAI	181 ± 93		
RPAI	98 ± 54	180 ± 68	0.844
LPAI	83 ± 49	110 ± 46	0.003
Patients with APC Intervention		70 ± 36	< 0.001
PAI	128 ± 52**		
RPAI	71 ± 33**	143 ± 38**	0.031
LPAI	57 ± 32**	89 ± 22*	0.006

^*^: *p* < 0.05, ** < 0.01: between patients with and without APCs

### Comparing Change in PA Index Between Patients Requiring APC Intervention and Those Not

The same analysis was performed in patients who underwent interventions for APCs at TCPC and those did not. In those with need for intervention a significant increase in PA index was observed between BCPS and TCPC (128 mm^2^/m^2^ to 143 mm^2^/m^2^, *p* = 0.031). On the contrary, when no intervention was indicated, the change in PA index was not significant (181 mm^2^/m^2^ to 180 mm^2^/m^2^, *p* = 0.844). Nonetheless, both groups showed considerable increase of right PA index. Left PA index did not show an increment, but a significant decrease in those without intervention (83 mm^2^/m^2^ to 70 mm^2^/m^2^, *p* < 0.001). When compared to each other, the two groups showed significant differences in PA index, right and left PA index, both at BCPS and TCPC (Table 3).

## Discussion

In the present study, we demonstrated that the occurrence of APCs prior to BCPS did not influence PA index at the time of BCPS. On the contrary, the presence of APCs before TCPC showed an association with a smaller PA index at the time of TCPC. A significant change in right and left PA index between BCPS and TCPC was observed in patients with APCs. Patients who underwent intervention for APCs also demonstrated a lower PA index at TCPC, as well as a significant change in PA index between BCPS and TCPS.

Various factors have been discussed to be associated with the development of APCs. In earlier studies, we showed that the development of APCs pre-BCPS was associated with the anatomical subtype of aortic atresia and mitral atresia (AA/MA) and shunt types of right ventricle to pulmonary artery conduit (RVPAC) in patients with HLHS after the Norwood procedure [13]. Grosse-Wortmann and colleagues proposed that APC may be an adaptive mechanism during prolonged periods of cyanosis, decreased volume and velocity of flow, or promoted by low blood flow to the lungs [14]. Our data supported this hypothesis because patients who had APCs at the time of TCPC were associated with smaller PA index at the time of BCPS, compared with those did not have APCs. The short-term increase in oxygen saturation after formation of APCs suggests that APC growth may be induced by hypoxia [2, 15]. However, empirical data do not suffice to verify the hypothesis and the need for molecular analysis is called for. Sandeep et al. approached the topic of biomarkers that influence APC growth but came to the conclusion that plasma factors and angiogenic activity correlate poorly with APC severity in patients with single ventricles, suggesting complex mechanisms of angiogenesis [16].

As for the relationship between PA index and the presence of APCs in single ventricle patients, Seger et al. found that PA index was the only variable associated with APC flow (size inversely related to APC flow) in patients who underwent pre-Fontan cardiac magnetic resonance (CMR) imaging [17]. Latus et al. showed that PA index was inversely correlated with APC flow and APC flow was associated with smaller PA size in patients after the Fontan procedure, suggesting that small PA may represent a stimulus for the development of APCs in Fontan patients [8]. Our results showed that presence of APCs was associated with underdevelopment of PA before TCPC but not with PA size before BCPS. While there is no correlation between PA index and APCs prior to BCPS, this changes after the procedure is performed. We assume that before BCPS patients had shunt-dependent parallel circulation, in which PA flow might be determined by anatomical factors, such as size of aortopulmonary shunt or degree of pulmonary stenosis. Therefore, PA size might not be correlated with pulmonary blood flow. After BCPS, SVC flow becomes the only source of the pulmonary blood flow, PA size might become a more representative parameter and correlate with the development of APCs. Hydrodynamic circumstances and acting pressures change after BCPS is completed which may lead to a stronger mutual influence between PA index and APC. It is of note that patients who had APCs before TCPC had smaller PA index not only at the time of TCPC but also at the time of BCPS. We think these data are strong evidence that small PA may represent a stimulus for the development of APCs. Further studies must clarify the cause-and-effect relations taking into account other possible impact factors.

A correlation between PA index and APCs has been described several times, but to our knowledge we are the first to evaluate and compare the changes of PA index in association with occurrence of APC, and over the entire period of Fontan palliation. Schmiel, et al. showed that the presence of APCs was associated with lower PA index at the time of TCPC [1]. Wang and colleagues reported a significant negative correlation between APC flow and the PA index in patients who underwent bidirectional Glenn shunting [18]. In this current study, we analyzed the change of PA index dependent on APCs. While there was no significant progression in PA index in neither the APC group nor the group without APCs, we could demonstrate right PA index increased significantly in patients with APCs. This was even more prominent in cases where patients had to undergo interventional closure of APCs. Our results suggested that development of APCs between BCPS and TCPC may be a compensation of small pulmonary arteries, and the size of pulmonary arteries increases in spite of the appearance of APCs. In other words, we assume that the development of APCs does not impair the growth of pulmonary arteries. In patients who developed APCs before TCPC, the right PA index increased significantly more than in those who did not. The left PA index tended to decrease in both patients with and without APCs, however the extent of the decrease was milder in patients with APCs compared to those without. This is a new finding. This is a new finding, and we speculate that an increase in the total pulmonary vascular beds between BCPS and TCPC might enable the development of central PA and APCs in these patients. Powell suggests that APCs may also increase PA pressure by adding to pulmonary blood flow [19]. APC blood flow may also compete with PA flow, likely via a rise in mean PA pressure [14]. Dori et al. reported that embolization of APCs resulted in significant decreases in total pulmonary blood flow as well as the ratio of pulmonary-to-systemic flow [20]. It might be possible that the increase in PA pressure may even trigger the growth of the pulmonary arteries. It is noteworthy that patients with a sufficiently large PA index did not experience an increase in PA index and did not develop APCs, whereas in patients with small PA index; PA index increased and they also developed APCs during the period between BCPS and TCPC. Further studies are mandatory to clarify the relationship of PA development and APC development, and their effects on the outcomes after the Fontan procedure. This study was designed to gain a deeper understanding of the postoperative period after the different stages of palliation. This should make it possible to interpret individual courses and to better assess possible complications that patients might expect. This contributes to a deeper understanding of the clinical management of the Fontan patient.

## Study limitations

This study is limited by its retrospective, non-randomized, and single-center design. The ability to identify APCs is highly dependent on technical variables and introduced detection bias. The time of the development of APCs may differ from the time of catheterization and may therefore introduce detection bias. Furthermore, quantitative analysis of APC burden was not available. Angiography is not routinely performed as a follow up after Fontan completion or APC closure. Further development of the PA index and whether the development might be influenced by interventional closure of the APCs could not be determined.

## Conclusions

Presence of APCs did not influence the PA size by the time of BCPS. However, presence of APCs was associated with underdevelopment of PA at TCPC, especially in patients who needed interventional closure of APCs.

## Abbreviations

APC: Aortopulmonary collateral
BCPS: Bidirectional cavopulmonary shunt
CI: Confidence interval
HLHS: Hypoplastic left heart syndrome
HR: Hazard ratio
ICU: Intensive care unit
IQR: Interquartile ranges
LIMA: Left internal mammary artery
OR: Odds ratio
PA: Pulmonary artery
RIMA: Right internal mammary artery
SVC: Superior vena cava
TCPC: Total cavopulmonary connection
